# Coronary Stent Abscess After Percutaneous Coronary Intervention: Diagnostic and Therapeutic Challenges

**DOI:** 10.1016/j.jaccas.2026.108308

**Published:** 2026-05-13

**Authors:** Sharath Nagesh, Ramya Das Nageri Kunnath, Pulugundla Varun, Sreevilasam P. Abhilash, Vivek Pillai, K.P. Dinoop, Harikrishnan Sivadasanpillai

**Affiliations:** a Department of Cardiology, Sree Chitra Tirunal Institute for Medical Sciences and Technology, Thiruvananthapuram, Kerala, India; b Department of Cardiovascular and Thoracic Surgery, Sree Chitra Tirunal Institute for Medical Sciences and Technology, Thiruvananthapuram, Kerala, India; c Department of Microbiology, Sree Chitra Tirunal Institute for Medical Sciences and Technology, Thiruvananthapuram, Kerala, India

**Keywords:** coronary pseudoaneurysm, coronary stent infection, fluorodeoxyglucose PET-CT, percutaneous coronary intervention, surgical management

## Abstract

**Background:**

Coronary stent abscess is a rare but potentially fatal complication of percutaneous coronary intervention (PCI), often presenting with nonspecific symptoms and leading to delayed diagnosis.

**Case Summary:**

We report a single-center experience of 5 patients with suspected coronary stent abscess, presenting after PCI with fever and recurrent chest pain. All cases demonstrated a coronary pseudoaneurysm on angiography. Blood cultures were positive in 3 patients. Advanced imaging, including computed tomography and coronary angiography, aided anatomical definition; however, positron emission tomography-computed tomography was nondiagnostic. Three patients underwent surgical intervention with stent removal and coronary artery bypass grafting, whereas one underwent percutaneous covered stent implantation. Two elderly patients with delayed presentation, including one with hemodynamic compromise, died despite aggressive management.

**Discussion:**

Coronary stent abscess remains a diagnostic challenge. Early suspicion in post-PCI patients with systemic symptoms supported by multimodality imaging and timely initiation of antibiotic therapy followed by surgical intervention may improve outcomes, particularly in high-risk populations.

**Take-Home Messages:**

Coronary stent infection should be suspected in post–percutaneous coronary intervention patients with pseudoaneurysm and systemic symptoms, even when microbiology and positron emission tomography imaging results are negative. Early recognition, prolonged targeted antibiotic therapy, and timely surgical intervention remain central to improving outcomes.

## Visual Summary

**Figure:**
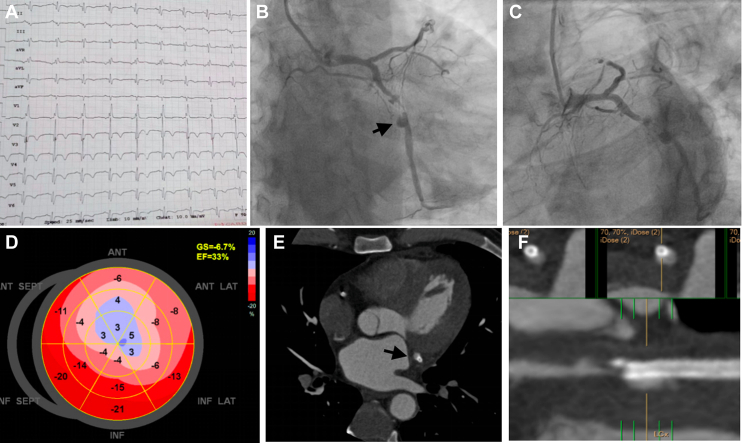

Percutaneous coronary intervention (PCI) with stent implantation has become the cornerstone of modern interventional cardiology. Despite widespread use of stents, coronary stent infection is extremely rare but can have devastating consequences.^1^ Despite aggressive interventions, the mortality rate remains high.^2^ We present a case series of 5 patients with suspected coronary stent abscess managed by different approaches, highlighting the diagnostic dilemma, therapeutic challenges, and variable outcomes associated with the condition.

**Take-Home Messages**

• Coronary stent infection should be suspected in post–percutaneous coronary intervention patients with pseudoaneurysm and systemic symptoms, even when microbiology and positron emission tomography imaging results are negative.
• Early recognition, prolonged targeted antibiotic therapy, and timely surgical intervention remain central to improving outcomes.

## Case 1

A 73-year-old man with long-standing diabetes mellitus and hypertension had previously undergone intravascular ultrasound –guided left main bifurcation PCI involving the left main, left anterior descending (LAD), and left circumflex (LCx) arteries using a minicrush technique (Video 1) with overlapping everolimus-eluting stents (left main-LAD artery: 4 × 18-mm XIENCE Sierra stent [Abbott], LAD artery: 3 × 33-mm XIENCE Sierra stent, LCx artery: 4 × 20-mm SYNERGY XD stent (Boston Scientific Corporation) stent). Two months later, he presented with recurrent high-grade fever, progressive weight loss (15 kg over 2 months), and episodes of angina at rest. Initially, pyrexia of unknown origin was suspected, and noncardiac infectious sources were excluded. Blood cultures subsequently grew *Staphylococcus aureus*.

Given the recent coronary intervention and recurrent angina, repeat coronary angiography was performed, which demonstrated aneurysmal dilation of the distal left main and proximal LAD arteries with impaired distal LAD artery flow (Video 2). Targeted intravenous antibiotics were initiated, and he was referred to our center. On admission, laboratory evaluation revealed leukocytosis with neutrophilia, elevated inflammatory markers, and hypoalbuminemia. Transthoracic echocardiography showed marked left ventricular systolic dysfunction, severe mitral regurgitation, and a large pseudoaneurysm involving the LAD artery stent. Computed tomography (CT) confirmed a large pseudoaneurysm extending across the stented segments of left main and LAD arteries. After discussion with a multidisciplinary heart team, surgical repair with coronary artery bypass grafting (CABG) was planned.

**Figure 1:**
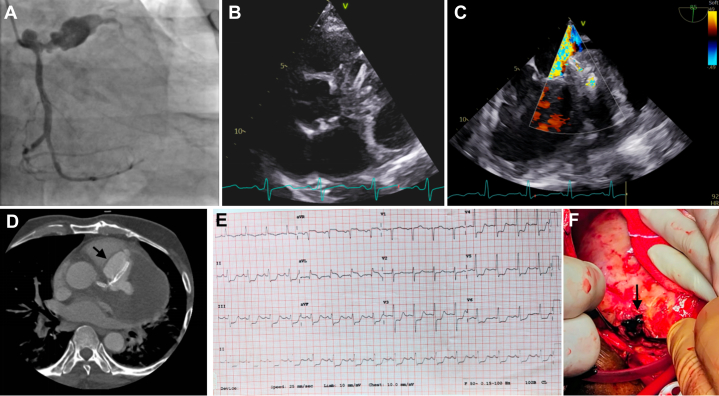
Case 1: Left Main-Left Anterior Descending Artery Pseudoaneurysm With Operative Correlation (A) Coronary angiography showing pseudoaneurysmal dilatation involving the distal left main and proximal left anterior descending artery. (B) Parasternal short-axis transthoracic echocardiographic view demonstrating the left anterior descending artery stent with surrounding pseudoaneurysm. (C) Midesophageal transesophageal echocardiographic view showing severe mitral regurgitation and flow into the involved left anterior descending artery segment. (D) Coronary computed tomography angiography demonstrating a large collection surrounding the stented segment of the left main-left anterior descending arteries (black arrow). (E) Electrocardiogram during in-hospital ischemic deterioration showing ST-segment elevation in aVL and ST-segment depression in inferior and lateral chest leads. (F) Intraoperative image showing the opened pseudoaneurysm cavity with purulent material (black arrow).

After 3 weeks of antibiotic therapy with partial clinical stabilization, he developed an in-hospital lateral-wall ST-segment elevation myocardial infarction with acute heart failure. In view of the large infected pseudoaneurysm, both thrombolysis and primary PCI were deemed high risk and deferred. He underwent emergency CABG with mitral valve repair, pseudoaneurysm repair, and removal of infected stent material. Intraoperatively, the proximal LAD artery was markedly dilated with a thick-walled cavity containing thrombus and purulent material. Representative angiographic, echocardiographic, CT, electrocardiographic (ECG), and intraoperative findings are shown in Figure 1. His postoperative course was complicated by severe sepsis with multiorgan dysfunction, acute kidney injury requiring renal replacement therapy, coagulopathy, and recurrent ventricular arrhythmias. Despite aggressive supportive measures, he died on postoperative day 4.

## Case 2

A 46-year-old man without prior comorbidities underwent PCI to LAD artery with sirolimus-eluting stents (3.5 × 20-mm and 2.75 × 20-mm PathFinder stents; Sahajanand Laser Technology Limited) for non–ST-segment elevation myocardial infarction at an outside center. One week later, he developed a persistent high-grade fever without an identifiable source. Repeat coronary angiography demonstrated a small pseudoaneurysm within the stented segment of the LAD artery. He was treated conservatively with a 2-week course of antibiotics; blood culture results from that admission were unavailable.

**Figure 2:**
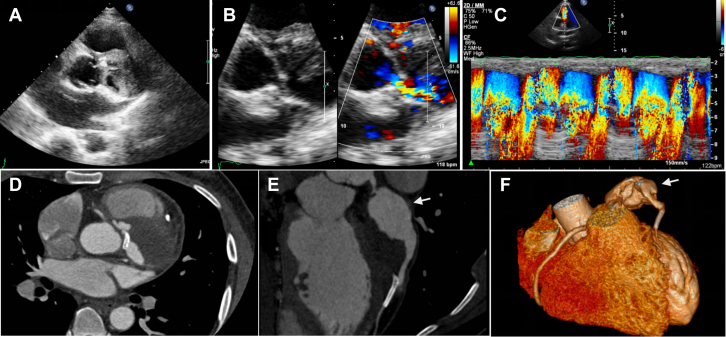
Case 2: Left Anterior Descending Infected Pseudoaneurysm With Right Ventricular Outflow Tract Communication (A and B) Parasternal short-axis echocardiographic views showing a pseudoaneurysmal cavity adjacent to the left coronary sinus and left main region. (C) Color M-mode image demonstrating continuous flow from the pseudoaneurysm into the right ventricular outflow tract. (D and E) Contrast-enhanced computed tomography images showing pseudoaneurysm arising from the stented left anterior descending artery segment. (F) Three-dimensional reconstructed computed tomography image showing the pseudoaneurysm in relation to the right ventricular outflow tract (white arrow).

Three months later, he developed a recurrence of fever spikes and was referred to our institute. Transthoracic echocardiography demonstrated a large echolucent structure adjacent to the aortic root, communicating with the right ventricular outflow tract (RVOT). Coronary CT angiography revealed a large nonenhancing collection surrounding the proximal LAD artery with a free-floating stent within the cavity and communicating with the RVOT, consistent with an infected pseudoaneurysm and abscess formation. Blood cultures grew *Pseudomonas aeruginosa*. Key echocardiographic and CT findings are shown in Figure 2.

The patient received 6 weeks of sensitivity-guided intravenous antibiotics, after which follow-up blood cultures were sterile. Following clinical stabilization, he underwent surgical pseudoaneurysm repair, stent retrieval, closure of communication with the RVOT, and CABG using the left internal mammary artery to the LAD artery. His postoperative course was uneventful, and he remains well on follow-up.

## Case 3

A 74-year-old man with diabetes, hypertension, and smoking history presented with inferior wall myocardial infarction and underwent PCI to the right coronary artery (RCA) (Video 3) with an everolimus-eluting stent (3 × 40-mm Tetrilimus stent; Sahajanand Medical Technologies). Two weeks later, he developed high-grade fever and recurrent chest pain. Repeat angiography revealed aneurysmal dilatation of the stented RCA segment (Video 4). Inflammatory markers were elevated, but blood cultures were sterile. Echocardiography showed preserved ventricular function with a small pseudoaneurysm adjacent to the aortic sinus. CT confirmed a 12 × 7-mm pseudoaneurysm surrounding the mid-RCA stent with adjacent hematoma; positron emission tomography-CT (PET-CT) result was negative.

In the absence of microbiologic confirmation and given negative metabolic imaging, a noninfective mechanical etiology was considered more likely at the time. However, the temporal association of fever followed by persistent ischemic symptoms and elevated inflammatory markers kept the possibility of an underlying infective process in consideration, although it could not be confirmed definitively.

**Figure 3:**
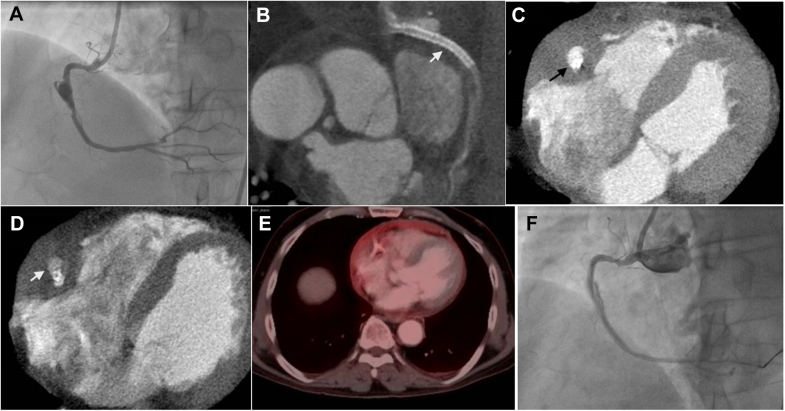
Case 3: Right Coronary Artery Pseudoaneurysm Treated With Covered Stent Implantation (A) Repeat coronary angiography after fever onset showing pseudoaneurysm arising from the stented right coronary artery segment. (B to D) Contrast-enhanced computed tomography images demonstrating the right coronary artery pseudoaneurysm (white and black arrows). (E) Fluorodeoxyglucose positron emission tomography-computed tomography showing no abnormal uptake at the pseudoaneurysm site. (F) Final angiogram after covered stent implantation showing exclusion of contrast filling into the pseudoaneurysm.

Because of the small pseudoaneurysm, stable hemodynamics, negative findings from blood cultures and PET-CT, and high surgical risk, he was treated with empiric antibiotics followed by covered stent implantation across the diseased segment. Angiography showed marked reduction of pseudoaneurysm filling, and the result was deemed satisfactory. The angiographic, CT, and PET-CT findings, together with the final angiographic appearance after covered stent implantatio, are shown in Figure 3. He was discharged in stable condition, but 2 months later, he suffered sudden cardiac arrest at home and died. Although a causal relationship cannot be established, this outcome raises the possibility that an unrecognized infective process may have contributed, highlighting the limitations of percutaneous exclusion strategies in cases where infection cannot be confidently excluded.

## Case 4

A 67-year-old woman with diabetes and hypertension presented with unstable angina and underwent PCI to RCA (Video 5) with 2 everolimus-eluting stents (3.5 × 23-mm and 3 × 33-mm XIENCE Xpedition stents; Abbott) at an outside center. Four days later, she developed recurrent chest pain without fever, and repeat angiography showed a small pseudoaneurysm within the stented RCA segment (Video 6). Blood culture grew *Staphylococcus hominis*, and appropriate intravenous antibiotics were given for 2 weeks and she was discharged.

**Figure 4:**
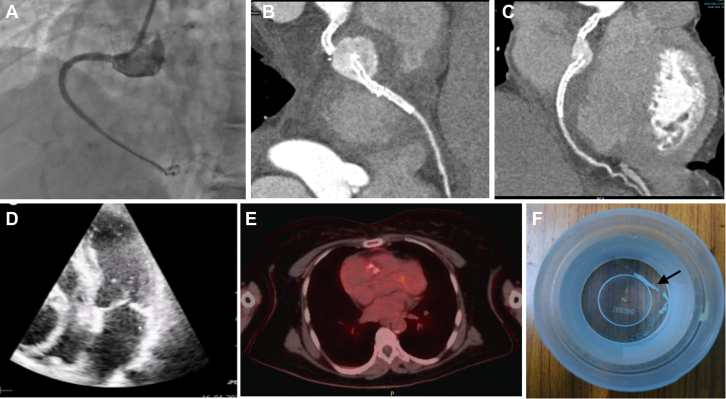
Case 4: Right Coronary Artery–Infected Pseudoaneurysm With Negative Positron Emission Tomography-Computed Tomography Result and Surgical Retrieval (A) Final angiographic result after index percutaneous coronary intervention at right coronary artery with TIMI flow grade 3. (B and C) Coronary computed tomography angiography after fever onset demonstrating pseudoaneurysm involving the stented right coronary artery segment with stent fracture. (D) Apical 4-chamber echocardiographic view showing a large pseudoaneurysm anterior to the right atrium. (E) Fluorodeoxyglucose positron emission tomography-computed tomography showing no significant uptake at the pseudoaneurysm site. (F) Retrieved stent material from the pseudoaneurysm cavity (black arrow).

One month later, she developed high-grade fever and worsening chest pain and was referred to our institute. Echocardiography showed mild left ventricular dysfunction and a large pseudoaneurysm adjacent to the right atrium. Coronary CT angiography with contrast medium demonstrated a 40 × 50-mm pseudoaneurysm arising from the distal RCA stent, with thickening and enhancement of the surrounding soft tissue and adjacent pericardium. PET-CT showed no significant uptake. Representative angiographic, echocardiographic, CT, PET-CT, and operative findings are shown in Figure 4.

Although *S hominis* was isolated, the possibility of blood culture contamination cannot be excluded given its known association with skin flora. However, the clinical course, characterized by recurrent episodes of chest pain and fever following PCI, together with progressive pseudoaneurysm formation and contrast-enhanced CT findings of perivascular and pericardial inflammation, raised a strong suspicion of an underlying infective process. Dense adhesions between the pseudoaneurysm and pericardium identified intraoperatively further supported an inflammatory or infective etiology. She received culture-directed antibiotics for 6 weeks followed by surgical stent retrieval, pseudoaneurysm repair, and CABG. Recovery was uneventful, and she remained asymptomatic on follow-up.

This patient was previously reported as an individual case report.^3^ The current article includes additional unpublished imaging and incorporates this case into a cumulative institutional analysis. No previously published text or figures have been reused.

## Case 5

A 58-year-old man with a history of inferior wall myocardial infarction and preserved left ventricular function underwent primary PCI to RCA (M'Sure-S drug-eluting stent [DES]; Relisys Medical Devices Limited), followed by staged PCI to the LAD artery with I-SYNC drug-eluting stent (Purple MicroPort Cardiovascular Pvt Ltd) and FIREHAWK drug-eluting stent (MicroPort Scientific Corporation), and to the obtuse marginal artery with an M'Sure-S drug-eluting stent (Relisys Medical Devices Limited). One week after the final PCI, he developed fever with chest pain, which was treated empirically with antibiotics and escalation of antianginal therapy. The fever resolved after 3 days, and no further evaluation was carried out. There was no recurrence of fever thereafter.

**Figure 5:**
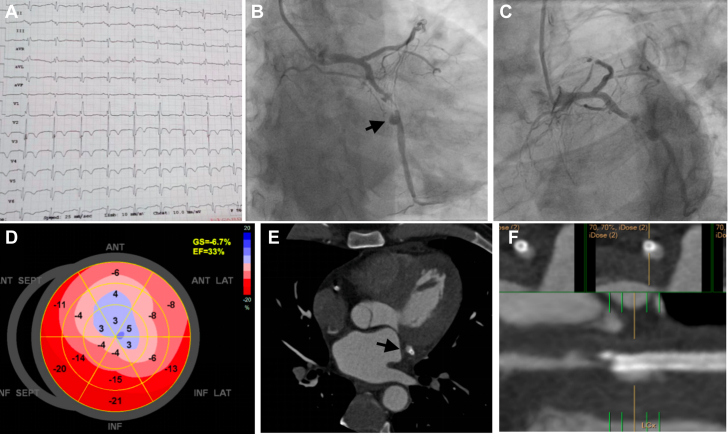
Case 5: Residual Pseudoaneurysm With Recurrent Ischemic Presentation (A) Electrocardiogram at presentation showing new right bundle branch block with anterior Q waves, consistent with evolved anterior wall myocardial infarction. Coronary angiography in anteroposterior caudal and left anterior oblique caudal projections showing a small pseudoaneurysm involving the obtuse marginal stent; the black arrow in panel B indicates the pseudoaneurysm. Note chronic total occlusion/in-stent restenosis of the distal left anterior descending artery. (D) Strain imaging demonstrating reduced global longitudinal strain with marked anterior, anteroseptal, and anterolateral dysfunction. (E and F) Coronary computed tomography angiography showing the pseudoaneurysm involving the obtuse marginal stented segment without radiological evidence of ongoing infection; the black arrow in panel E indicates the pseudoaneurysm.

Three months later, he presented to our institute with a history of chest pain lasting 2 days. ECG showed features of evolved anterior wall myocardial infarction with new-onset Q waves and right bundle branch block in the anterior leads. Troponin was markedly elevated. Coronary angiography revealed a pseudoaneurysm involving the obtuse marginal stent and in-stent restenosis of the LAD artery. Echocardiography demonstrated new-onset severe left ventricular systolic dysfunction with anterior and anteroseptal hypokinesia and mild mitral regurgitation. Given the prior history of fever and the presence of a pseudoaneurysm, stent-related infection was initially considered. Coronary CT angiogram confirmed a small pseudoaneurysm over the LCx artery stent with no evidence of ongoing infection (perivascular thickening or enhancement). Inflammatory markers were normal (except for mildly elevated C-reactive protein), and blood cultures were sterile. ECG, angiographic, echocardiographic, and CT findings are shown in Figure 5.

Given the absence of persistent systemic symptoms, negative microbiologic workup, and lack of imaging features suggestive of infection, a diagnosis of stent-related infection was considered less likely. The pseudoaneurysm was therefore interpreted as a probable noninfective mechanical complication, and antibiotic therapy was not initiated. He was managed medically with anticoagulation, antiplatelet, and optimized antianginal therapy and was planned for elective CABG with pseudoaneurysm repair on follow-up. He experienced no recurrence of chest pain since then. This case highlights that the presence of a post-PCI pseudoaneurysm does not necessarily imply infection and underscores the importance of careful clinical and investigative correlation to avoid overdiagnosis and unnecessary treatment.

The salient clinical, microbiologic, management, and outcome features of all 5 cases are summarized in Table 1.

**Table 1:** Clinical Characteristics, Microbiologic Findings, Management, and Outcomes of 5 Patients With Suspected Coronary Stent Abscess

	Case 1	Case 2	Case 3	Case 4	Case 5
Age, y	73	46	74	67	58
Sex	Male	Male	Male	Female	Male
Comorbidities	Diabetes, hypertension	None	Diabetes, hypertension	Diabetes, hypertension	None
Indication for index angioplasty	Non–ST-segment elevation myocardial infarction	Non–ST-segment elevation myocardial infarction	Inferior wall myocardial infarction	Unstable angina	Inferior wall myocardial infarction
Stent type	Everolimus-eluting	Sirolimus-eluting	Everolimus-eluting	Everolimus-eluting	Sirolimus-eluting
Stent model	XIENCE Sierra	PathFinder	Tetrilimus	XIENCE Xpedition	M'Sure-S
Manufacturer	Abbott	Sahajanand Laser Technology Limited	Sahajanand Medical Technologies	Abbott	Relisys Medical Devices Limited
Vessel affected	LM, LAD	LAD	RCA	RCA	LCx
Time interval between PCI and initial presentation to hospital	2 mo	1 wk	2 wk	4 d	3 mo
Presenting symptom	Fever, chest pain	Fever	Fever, chest pain	Fever, chest pain	Fever, chest pain
Hemodynamic compromise or heart failure	Yes	No	No	No	No
LV function	Moderate LV dysfunction	Good LV function	Good LV function	Mild LV dysfunction	Severe LV dysfunction
Pseudoaneurysm	Yes	Yes	Yes	Yes	Yes
Blood culture	*Staphylococcus aureus*	*Pseudomonas aeruginosa*	Sterile	*Staphylococcus hominis*	Sterile
Diagnosis of stent abscess	Definite	Definite	Possible	Probable	Unlikely
Intervention	Pseudoaneurysm repair + stent retrieval + Mitral valve repair + CABG	Pseudoaneurysm repair + stent retrieval + CABG	Covered stent implantation	Pseudoaneurysm repair + stent retrieval + CABG	Planned elective pseudoaneurysm repair + stent retrieval + CABG
Outcome	Died	Survived	Died	Survived	Survived

CABG = coronary artery bypass grafting; LAD = left anterior descending; LCx = left circumflex; LM = left main; LV = left ventricular; PCI = percutaneous coronary intervention; RCA = right coronary artery.

## Discussion

Coronary stent abscess is an extremely rare but potentially devastating complication of PCI. Although uncommon, reported mortality remains high, largely due to delayed recognition and advanced disease at presentation.^4^^,^^5^ The most common organism implicated is *S aureus*, particularly methicillin-resistant *S aureus*, because of its propensity for adherence to foreign material and biofilm formation.^1^ Presentation can be varied. In our series, patients presented across a broad clinical spectrum, highlighting that the principal challenge is often diagnostic rather than therapeutic. One patient received a diagnosis of pyrexia of unknown origin after an extensive workup. The earliest manifestations are frequently nonspecific. Fever after PCI may be transient or attributed to noncardiac causes, and recurrent chest pain may be interpreted as routine postprocedural angina. Some patients improve temporarily with a short course of empirical antibiotics, which may further obscure the diagnosis. This variability highlights the need for a high index of suspicion in any patient who underwent recent PCI and develops persistent fever, unexplained inflammatory markers, constitutional symptoms, or recurrent ischemia.

Formation of pseudoaneurysm is the most important imaging clue in suspected cases. However, a post-PCI pseudoaneurysm does not, by itself, confirm infection because it may result from mechanical vessel injury or a contained perforation. Conversely, once stent infection becomes clinically significant, it commonly manifests with pseudoaneurysmal or cavitary destruction of the vessel wall.^4^^,^^5^ Therefore, pseudoaneurysm in the setting of systemic or inflammatory features should prompt active exclusion of infection. Treating an infected pseudoaneurysm conservatively as a sterile mechanical complication may lead to catastrophic consequences. Both patient-related and procedural factors may predispose to stent abscess formation (Table 2). Strict adherence to aseptic technique during PCI—including careful catheter handling, avoidance of hardware reuse, and minimization of prolonged sheath dwell time—remains the most effective preventive strategy against coronary stent infection.

**Table 2:** Patient-Related and Procedural Risk Factors for Coronary Stent Abscess

Procedure Related	Patient Related
Reuse of hardware like wires and catheters	Advanced age
Failure to maintain aseptic precautions	Diabetes mellitus
Local site infection	Chronic kidney disease
Procedural complications like hematoma/pseudoaneurysm formation	Malnutrition
Repeated use of the same access	Immunocompromised
Prolonged placement of arterial sheaths	
Complex PCI (bifurcation, CTO, calcific lesions)	

CTO = chronic total occlusion; PCI = percutaneous coronary intervention.

Diagnosis relies on clinical judgment supported by microbiology and imaging rather than any single definitive test. Blood cultures are valuable but may be negative, particularly after prior antibiotic exposure.^4^ In our series, microbiologic confirmation was not universal, highlighting that prior antibiotic exposure, intermittent bacteremia, or localized infection can reduce culture yield. Inflammatory markers are supportive but nonspecific. Coronary angiography often demonstrates aneurysmal dilation, irregular stent contours, or impaired distal flow, whereas coronary CT angiography provides superior anatomical definition for procedural planning and may show findings supportive of infection (eg, perivascular fat stranding). Fluorodeoxyglucose PET-CT has been used in vascular prosthetic infections, but its sensitivity in coronary stent abscess appears variable.^4^ All patients in our series who underwent PET-CT had negative results. False-negative PET results may occur because of small lesion size, cardiac motion, myocardial uptake, or prior antibiotics. A negative PET study should therefore be interpreted cautiously and should not exclude infection when clinical and structural findings are suggestive. Importantly, our series demonstrates that post-PCI pseudoaneurysm represents a spectrum ranging from mechanical injury to definite infection.

Management poses a separate challenge. Short-course or empirical antibiotic therapy may suppress systemic symptoms without eradicating infection, particularly in the presence of a biofilm on metallic stent material.^5^ In our series, early symptomatic improvement after empirical therapy did not always indicate resolution of the underlying process. In one patient, fever settled after early antibiotics, yet structural disease later persisted. In another, an apparent initial improvement was followed by recurrence and progression to a larger abscess cavity with a floating stent. Prolonged culture-directed antibiotic therapy is therefore essential in suspected or confirmed cases. However, antibiotics alone may be insufficient when infected hardware remains in situ.

Surgical intervention remains the most definitive treatment in operable patients.^4^^,^^5^ Removal of infected stent material, repair of the pseudoaneurysm, debridement of infected tissue, and CABG provide true source control. In our experience, patients who received prolonged antibiotic therapy followed by surgery had favorable outcomes once they survived the immediate postoperative period. Percutaneous management with covered stent implantation has been described as an alternative strategy in selected cases.^6^^,^^7^ The theoretical benefit is immediate exclusion of the pseudoaneurysm and avoidance of open surgery, particularly in high-risk patients. However, this approach does not remove infected material and therefore does not provide true source control. Persistent infection, recurrence, and late complications have been reported after percutaneous exclusion of the infected coronary segments.^6^^,^^7^ In our series, case 3 highlights that in the setting of diagnostic uncertainty, percutaneous exclusion may be inadequate if an underlying infective process is present but unrecognized. For this reason, covered stents should be considered primarily in carefully selected patients with prohibitive surgical risk after completion of antibiotics or as a bridging strategy rather than as definitive therapy.

One of the most challenging clinical scenarios in our series was the occurrence of in-hospital ST-segment elevation myocardial infarction in the setting of a known infected pseudoaneurysm. Standard reperfusion strategies become considerably complicated in this context. Thrombolysis carries a theoretical risk of aneurysm expansion or rupture, while repeat PCI in an infected and structurally compromised segment may exacerbate vessel injury and does not address the underlying infectious source. In such situations, urgent surgical intervention may represent the most definitive strategy, although operative risk is often elevated because of concurrent sepsis, ventricular dysfunction, and hemodynamic instability. This intersection of acute ischemia and active infection highlights the importance of early diagnosis before progression to complex and high-risk therapeutic dilemmas.

Outcome appears strongly influenced by timing and host factors.^8^ Delayed presentation allows progression to large pseudoaneurysm, recurrent infarction, ventricular dysfunction, and systemic instability, which increases perioperative risk. In our series, adverse outcomes were seen predominantly in elderly diabetic patients and in those presenting late or with hemodynamic compromise. Also, case 5 illustrates that not all post-PCI pseudoaneurysms indicate infection, and misclassification could result in unnecessary antibiotic treatment or invasive procedures.

In summary, coronary stent abscess poses a major diagnostic challenge. Early suspicion in patients with recent PCI, careful evaluation for pseudoaneurysm, cautious interpretation of negative cultures or PET imaging, and timely surgical source control are central to improving outcomes in this rare but serious complication.

## Conclusions

Coronary stent abscess should be considered in any patient who underwent recent PCI and develops fever, recurrent ischemia, or a new pseudoaneurysm, even when blood cultures are sterile or the result of PET imaging is negative. However, not all post-PCI pseudoaneurysms are infective, and careful clinical, microbiologic, and imaging correlation is essential to avoid misclassification. Temporary clinical improvement with empirical antibiotics does not exclude persistent infection, particularly when infected hardware remains in situ. Prolonged targeted antimicrobial therapy and timely surgical source control remain the most reliable management strategy, whereas percutaneous exclusion should be reserved for carefully selected high-risk patients. Early recognition and multidisciplinary decision-making are essential to interrupt disease progression and to improve survival in this challenging and often under-recognized complication.

**Visual Summary:**
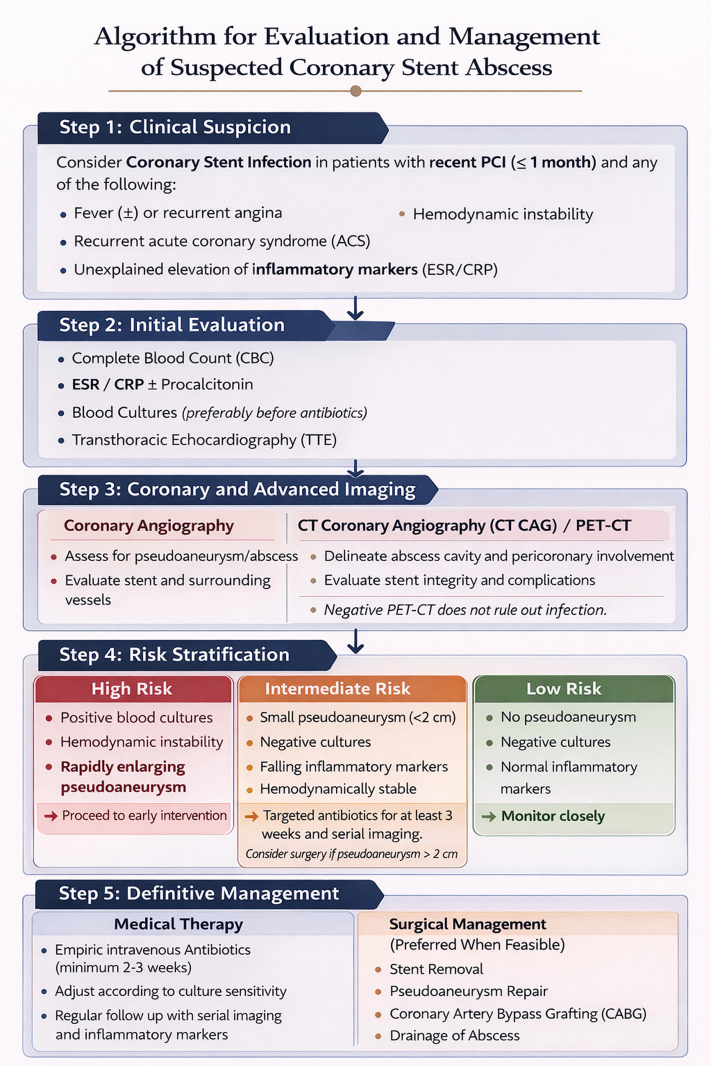
Proposed Diagnostic and Management Pathway in Coronary Stent Abscess Patients with recent PCI presenting with fever, recurrent ischemia, or new pseudoaneurysm should undergo structured evaluation including blood cultures and coronary imaging. Negative PET imaging result does not exclude infection. Prolonged targeted antimicrobial therapy and timely surgical source control represent the preferred management strategy, with percutaneous exclusion reserved for selected high-risk patients. CRP = C-reactive protein; CT = computed tomography; ESR = erythrocyte sedimentation rate; PCI = percutaneous coronary intervention; PET = positron emission tomography.

## Funding Support and Author Disclosures

The authors have reported that they have no relationships relevant to the contents of this paper to disclose.

## Appendix

Video 1 Coronary Angiography in Anteroposterior Caudal View Following Index PCI Demonstrating Satisfactory Stent Deployment in the Left Main Bifurcation Involving the Left Anterior Descending and Left Circumflex Arteries (Case 1)

Video 2 Repeat Coronary Angiography in Anteroposterior Caudal View Performed at Presentation with Clinical Suspicion of Stent Infection Demonstrating Aneurysmal Dilatation Involving the Left Main and Proximal Left Anterior Descending Artery With Impaired Distal Flow (Case 1)

Video 3 Coronary Angiography in Left Anterior Oblique Cranial View Following Index PCI Demonstrating Satisfactory Stent Deployment in the Right Coronary Artery (Case 3)

Video 4 Repeat Coronary Angiography in Left Anterior Oblique Cranial View Demonstrating Aneurysmal Dilatation of the Stented Right Coronary Artery Segment Consistent With Pseudoaneurysm Formation (Case 3)

Video 5 Coronary Angiography in Left Anterior Oblique Cranial View Following Index PCI Demonstrating Satisfactory Stent Deployment in the Right Coronary Artery (Case 4)

Video 6 Repeat Coronary Angiography in Left Anterior Oblique Cranial View Demonstrating a Pseudoaneurysm Arising From the Stented Right Coronary Artery Segment (Case 4)
